# Music in Displacement: Communal Gatherings as Anchors of Resilience for Older Persons

**DOI:** 10.1093/geroni/igaf122.1973

**Published:** 2025-12-31

**Authors:** Assaf Suberry, Liat Ayalon

**Affiliations:** Bar-Ilan University, Ramat-Gan, HaMerkaz, Israel; Bar-Ilan University, Ramat-Gan, HaMerkaz, Israel

## Abstract

This study aims to explore how musical gatherings serve as a source of psychological support for displaced older Israelis following the October 7, 2023, Hamas attacks. After 60,000 Israeli citizens were relocated to safer and more remote locations, musicians and artists generously held weekly musical performances. Additionally, community initiatives organized a variety of traditional and innovative events and activities. Through thematic analysis of 17 semi-structured interviews, participants describe these gatherings as temporary “bubbles in time” that provide emotional relief and social reconnection. The findings highlight that these gatherings not only offered a brief escape from daily hardships but also enabled the continuation of traditional ceremonies in an intergenerational setting, preserving cultural continuity despite displacement. Within these shared experiences, a sense of communitas emerged, reinforcing social bonds and collective resilience. Given their profound impact, communal gatherings should be integrated into crisis response protocols to support the well-being of displaced older persons.

